# Development and Preliminary Validation of the Physical Education-Study Process Questionnaire : Insights for Physical Education University Students

**DOI:** 10.3389/fpubh.2022.856167

**Published:** 2022-03-17

**Authors:** Amayra Tannoubi, Noomen Guelmami, Tore Bonsaksen, Nasr Chalghaf, Fairouz Azaiez, Nicola Luigi Bragazzi

**Affiliations:** ^1^Department of Human and Social Sciences, Higher Institute of Sport and Physical Education of Kef, University of Jendouba, Jendouba, Tunisia; ^2^Group for the Study of Development and Social Environment (GEDES), Faculty of Human and Social Science of Sfax, Sfax, Tunisia; ^3^Department of Health Sciences, Postgraduate School of Public Health, University of Genoa, Genoa, Italy; ^4^Department of Human Sciences, Higher Institute of Sport and Physical Education of Sfax, University of Sfax, Sfax, Tunisia; ^5^Department of Health and Nursing Science, Faculty of Social and Health Studies, Inland Norway University of Applied Sciences, Elverum, Norway; ^6^Department of Health, Faculty of Health Studies, VID Specialized University, Sandnes, Norway; ^7^Department of Mathematics and Statistics, York University, Toronto, ON, Canada; ^8^Department of Neuroscience, University of Genoa, Genoa, Italy

**Keywords:** approach to learning, deep approach, physical education, surface approach, psychometric

## Abstract

**Background:**

The Revised Two Factor Study Process Questionnaire (R-SPQ-2F) is used to examine students' study approaches in higher education. The questionnaire is designed to measure two factors: deep and surface approaches. In order to measure these approaches for students in physical education and sport, a new measurement instrument should take into consideration the practical context of this field of education that makes it specific to other fields.

**Objective:**

The present study aims (a) to develop and empirical test of a new instrument for measuring the study process in physical education and sports students, and (b) to test psychometric properties of the tool.

**Methods:**

Two exploratory and confirmatory samples of physical education students enrolled in a bachelor's degree program in physical education at the High Institute of Physical Education and Sports of Kef-Tunisia, aged 19–26 years, were recruited online among female students (*n* = 414) and male students (*n* = 393). The participants filled in Google Form survey including Physical Education-Study Process Questionnaire (PE-SPQ) and the Arabic version of the Revised Study Process Questionnaire-2 Factors (R-SPQ-2F).

**Results:**

Exploratory factor analysis showed a suitable four factors solution, which is approved by confirmatory factor analysis indices [χ2 = 466.47, TLI = 0.94, CFI = 0.95; RMSEA = 0.56 IC 90% (0.050–0.062)]. Internal consistency of the PE-SPQ simultaneously checked by McDonald's ω, Cronbach's α and Gutmann's λ6 showed good reliability of the PE-SPQ. Convergent validity examined by Average variance extracted (AVE) was good. The comparison between the AVE root mean square and Pearson correlation coefficients of each factor with his indicators reveals the discriminant validity of the PE-SPQ. Furthermore, Pearson's correlation between the PE-SPQ factors and the R-SPQ-2F establishes the concurrent validity of the new scale.

**Conclusion:**

The PE-SPQ scale is valid and reliable and can be used to assess study process factors in physical education students.

## Introduction

In recent years, the learning process in secondary and higher education has been the subject of research in its proper context. Indeed, understanding the learning process is necessary to increase the quality of learning. Therefore, it is crucial to have a comprehensive view of the student learning process in education (1). In fact, studies have focused on finding ways of explaining some of the main differences in students' approach to learning (2, 3).

In higher education, quality of teaching and learning are as yet topics of debate (4). Moreover, learning approaches adopted by students are common concerns in different fields such as business, nursing, and psychology (5–9). Students do not always adopt the learning approach best suited to bring about desired academic achievements and success. The academic achievement and success seem to vary depending on these approaches (10, 11). Also, learning approaches are different processes by which a student obtains, assimilates, and retains knowledge (12). Indeed, the learning approach denotes the student's overall pattern of study behaviors and attitudes in a given learning context (13).

Hailikari and Parpala (14) specified that learning approaches represent the management of the study tasks by students. Two distinct learning approaches are commonly reported, referred to as the “*deep approach” (DA)*, centered on comprehension of course material and seeking to relate ideas; and the “*surface approach” (SA)*, driven by rote learning without self-reflection (15). The distinction between DA and SA is particularly useful for academics who want to understand their students' learning and create a suitable educational environment (2, 16). Previous studies reported that an adequate class climate encourages students to use a deep approach to learning (17–19).

In order to measure these learning approaches, researchers have developed several questionnaires (20), such as the “Approaches and Study Skills Inventory for Students (ASSIST)” (21) or the “Learning and Studying Questionnaire” (22). However, one of the most widely used instruments to measure student-learning approaches (2), is the “Study Process Questionnaire” [SPQ; (23)] which was later revised to “Revised Study Process Questionnaire-2 Factor” [R-SPQ-2F, (24)]. The validity of this scale has been confirmed by various studies (25–32). Also, the R-SPQ-2F has been investigated in several countries, such as the USA (26), Japan (27), Ghana (33) and India (34). The tool confirmed its robustness to operationalize the DA and SA concepts in various fields of higher education, such as biology (32), dental medicine (35), orthopedics (36), and business (27). Likewise, different educational studies suggested that R-SPQ-2F scores were associated with personality, knowledge acquisition, academic performance, learning style preference, self-efficacy, goal orientation, and self-regulation strategies (37, 38).

It seems that to date, Physical education (PE) is a specific educational context with very distinct features, or something similar, and then outline how this discipline is distinct from others. The PE students must have theoretical knowledge in the humanities and social sciences, educational sciences, statistics, and biological and movement sciences (39, 40). Besides that, there is also practical training in individual sports, team sports and Martial arts (41, 42). Furthermore, in this field, the process of student training and learning involves several interdisciplinary (43) and transdisciplinary fields (44), and these students are able to become coaches and physical education teachers (45).

Several previous studies have noted future physical education will differ from education in other disciplines with regards to the physical structure of the classroom, characteristics of teaching content, professional tasks, and the status of the subject matter and teachers (46, 47).

Therefore, PE learning requires particular curriculum and tasks to prepare students for their careers: theoretical concerns, physical performance, technical skills acquisition, and practical knowledge.

PE students' learning approaches, may be related to this specific context. Indeed, the motivation of students in PE generally decreases after the first years. However, practical classes within academic PE settings are typically held in open spaces such as gymnasiums, tracks, and playgrounds, which will encourage students to view these classes as places to release their excess energy (48). As a result, concept measurements such as, satisfaction, autonomy, motivation, engagement and grit in learning of PE students is different from other students (49–52).

In relation to assessment, students who adopt a SA in the various subjects aim toward an accurate reproduction of the course (15). However, in PE the SA is linked to a practical component (e.g., proper execution of the sport movement). Also, the assessment of “theoretical knowledge” for PE students was conducted in a standard way, as in other more established subjects, by examinations, dissertations or multiple-choice questions (53). In contrast, the assessment of “practical knowledge” was less easy to achieve. Various assessment tools were developed, such as the use of motor skill and fitness tests, and point tables for performance in areas such as gymnastics, swimming and athletics.

However, hands-on PE classes may involve activities that are dangerous and characterized by its varied and vigorous aspect (54), such as contact sports or gymnastics (55), furthermore, the specificity of this environment may lead to safety-related incidents or issues compared to regular classrooms (56), such as injuries (57), sprains (58) or back pain (59). In addition, the PE student faces a range of contextual factors, that have the potential to present significant emotional demands (60, 61). These need to be addressed in the student's curriculum in order to prepare them to implement contextually relevant instructional content. It is also important to note that learning in an environment where theoretical principles and practical applications are closely related helps future PE teachers bridge the gap between theory and practice and generate theory from practice (62). Depending on the students' involvement within a practice or theorical learning activity a surface approach or a deep approach (63) four possibilities can arise: Deep Theoretical Task (DTT), Surface Theoretical Task (STT), Deep Practical Task (DPT) and Surface Practical Task (SPT).

All these features may distinguish the physical education student from other students in different fields of study. However, to the best of our knowledge, there is no instrument to measure study approaches in the specific context of physical education. Given the importance of the concept of study approach to learning in educational settings, the aim of the present study is (a) to develop a new instrument: Physical Education Study Process Questionnaire (PE-SPQ) for measuring the study process in physical education and sports, and (b) to test the psychometric properties of the instrument in terms of factor structure, validity and reliability.

## Materials and Methods

### Declaration of Ethics

This study has received the approval of the Ethics Committee of the “*High Institute of Sport and Physical Education, Kef, University of Jendouba, Jendouba, Tunisia*,” the “*High Institute of Sport and Physical Education of Sfax*” and the “*High Institute of Sport and Physical Education of Gafsa*.” The research was also approved by the Ethics Committee of the “*University of Jendouba*” and was undertaken in accordance with the legal standards of the “*Declaration of Helsinki 1964*” and its corresponding amendments.

Each participant was asked to complete the questionnaires after receiving an informed consent form. They were informed that there was no obligation to participate in the study, and that any refusal did not have to be justified. The study was described as a study of the vagaries of school life, without specifying the concepts of commitment to limit response bias.

### Participants and Data Collection

A sample of physical education students (*n* = 807) were recruited online. No exclusion criteria were used. The participants were enrolled in a bachelor's degree program in physical education at the High Institute of Physical Education and Sports of Kef-Tunisia.

Participants were invited to take part in the study through social media: Facebook (official page of the institute) and e-mail. An electronic survey was administered using the online survey portal, *Google forms®* (Online survey services), provided by *Google Inc (Google, California, USA)*, which is a cloud-based data management tool used universally to design and develop online questionnaires. This tool collects the email addresses of survey participants. By activating this option, each subject will only be limited to submitting one answer.

The age of the subjects varied between 19 and 26 years. Mean age was 21.82 ± 1.51 years. The proportion of female participants (*n* = 414, 51.3%) was similar to that of men (*n* = 393, 48.7%). The subjects recruited for the study were divided into two groups to conduct both exploratory and confirmatory studies.

A. Exploratory data were collected from 226 students aged 19–25 years (M = 21.90 ± 1.35). The subjects were recruited from both sexes, women (*n* = 101; 44.69%) and men (*n* = 125; 55.30%).
B. Confirmatory data were collected from a total of 581 students aged 19–26 years (M = 21.79 ± 1.57). The subjects were male (*n* = 268; 46.12%) and female (*n* = 313; 53, 87%).

### Instruments

#### Study Variables

In our study, the variables of gender and age were considered as basic demographic characteristics.

#### Arabic Version of the Revised Two-Factor Study Process Questionnaire (R-SPQ-2F)

This version was adapted by Khine and Afari (31) among students from a teachers' college in Abu Dhabi, UAE. The internal consistency (Cronbach alpha) for the 20-item questionnaire was considered acceptable reliability. Factor one, the deep approach (DA) with 10 items provided a Cronbach's alpha of 0.81, factor two, the surface approach (SA) with 10 items was 0.76, which were considered acceptable. The results were above the acceptable level of 0.70 for a scale consistency test as suggested by Hair et al. (64) and DeVellis and Thorpe (65). The Cronbach alpha reliability results for are comparable somewhat with the Cronbach alpha values 0.73 and 0.64 as reported by the developers of the R-SPQ-2F.

#### Physical Education Study Process Questionnaire PE-SPQ

The “Physical Education-Study Process Questionnaire” (PE-SPQ) was developed through a series of meetings among university teachers in educational sciences of pedagogical studies. They explored existing scales in the literature in relation to study processes (66–69). Also, the same procedure has been followed for the physical education context (70–74). However, given the theoretical and practical nature of PE training and assessment (75, 76), it was necessary to generate a pool of items that takes this specificity into account. Indeed, students in this field may opt for a deep approach to the content of practical subjects, while they may operate using a surface approach to academic knowledge, and vice versa. Similarly, the student may choose different approaches to studying in practical and theoretical courses. The task of the committee charged with this study led to the elaboration of a questionnaire of 20 items, allowing to measure the study process through four dimensions [Deep Theoretical Task (DTT) and Surface Theoretical Task (STT) Deep Practical Task (DPT) and Surface Practical Task (SPT)], each of which includes five items [example: *I am often interested in reviewing the information provided in the theoretical courses (DTT)/ I manage my theoretical courses by repetition, and go over them several times until I memorize them without understanding the content (STT)/ I do my best in the practical courses because I find these sessions interesting (DPT)/ One of my goals is to pass the practical exams with as little effort as possible (SPT)]*.

At this point, we were able to identify the key aspects that characterize the dimensions of the study process. In addition, we tried to integrate the specific characteristics of the study population into the items. When writing the items, we chose clearly comprehensible and unambiguous vocabulary. The recommendations were to generate standard items that are not specific to a particular environment and valid for studying the study process in PE students around the world.

Five female and male university teachers/researchers (two professors and three associate professors) with ages ranging from 41 to 48 years old made up a focus group. They devoted at least 15 years to all of their scientific and educational endeavors. Among the group are two of the manuscript's authors.

The focus group discussed to identify potential difficulties, which could pose problems related to the cultural context. The wording of the items that posed potential problems was then revised during the discussion. Finally, a pre-test of the paper version of the questionnaire was conducted on a group of females (*n* = 28) and males (*n* = 24) students to assess item comprehension.

Students participating in the survey responded to each item by choosing a categorical frequency response on a five-point Likert scale ranging from 1 (response A) to 5 (response E) (A: *never or rarely true for me*; B*: true for me occasionally*; C: *true for me every other time*; D: *often true for me*; E*: always or almost always true for me*).

### Statistical Analysis

Statistical analyses were performed using IBM SPSS version 26 (IBM Corp., Armonk, NY, USA), Lavaan package in RStudio and the free JASP 2020 software.

Preliminary analysis of the numerical data was carried out to examine the quality of the data collected and inspect for anomalies or missing boxes. Subsequently, univariate (Skewness and Kurtosis) and multivariate normality tests using the Mardia coefficient were performed and descriptive statistics for each variable were completed.

Exploratory factor analysis was performed by the Unweighted Least Squares method with Direct-Oblimin rotation and Kaiser normalization. Factor analysis was performed if KMO >0.80 and a significant Bartlet test's Chi-square (77).

The reliability of the instrument was examined simultaneously by the Cronbach coefficient α, the McDonald coefficient ω and the Gutmann's coefficient λ6.

A Cronbach's α above the threshold of 0.70 was considered as acceptable, above 0.80 as good, and between 0.90 and 0.95 as excellent). The questionnaire structure for the entire population was carried out by confirmatory factor analysis (CFA). For the McDonald coefficient ω and the Gutmann's coefficient λ6, Values above 0.70 were considered appropriate (78–80).

The confirmatory factorial analysis was conducted by the robust Diagonally Weighted Least Squares (DWLS) method (81). According to Comrey and Lee (82), the robustness of an indicator, in CFA, is demonstrated with his high factor loadings. Thresholds suggest that a factor loading > 0.71 is considered excellent, > 0.63 is considered very good, > 0.55 is considered acceptable and < 0.45 is considered poor.

Several CFA indices were used to examine the model: (1) the χ2; (2) χ2/DF, (3) the Comparative Fit Index (CFI); (4) the Tucker-Lewis Index (TLI); (5) Standardized Root Mean Square Residual, and (6) the Root Mean Square of error Approximation RMSEA.

Hu and Bentler (83) suggested values >0.95 for the CFI and TLI, and RMSEA values <0.08 for reasonable adjustments.

Convergent validity and discriminant validity were assessed, respectively, by calculating the average variance extracted (AVE) and comparing the square roots of the AVE values to the correlation coefficients between latent constructs (84). Discriminant validity is demonstrated when the variance shared by two different latent constructs is less than the variance shared by that variable and its indicators. This implies that the square root of the AVE must be greater than all correlations between latent constructs.

Concurrent validity was examined by Pearson's correlation between the scores of the four scale factors and the scores measured on the Arabic R-SPQ-2F.

## Results

### Normality and Descriptive Statistics

Statistical analysis began with the calculation of descriptive statistics (Table 1) (means and standard deviations) and inspection of the distributions of the 20 questionnaire items. For each item, the distribution appeared to be normal given the measures of kurtosis [−2, 2] and skewness [−3, 3].

**Table 1:** Descriptive statistics, normality coefficients and Lambda factor loadings of the PE-SPQ.

	**Mean**	**Std. deviation**	**Skewness**	**Kurtosis**	**Lambda**
I1	3.80	1.11	−0.74	−0.19	0.93
I5	3.97	0.93	−0.69	0.13	0.87
I9	3.82	1.04	−0.55	−0.36	0.86
I13	3.66	1.02	−0.45	−0.19	0.84
I17	3.79	1.09	−0.69	−0.08	0.83
I2	3.37	1.28	−0.36	−0.93	0.86
I6	3.03	1.21	−0.06	−0.90	0.85
I10	2.83	1.22	0.15	−0.88	0.84
I14	2.80	1.28	0.19	−1.00	0.83
I18	2.99	1.24	−0.05	−0.86	0.67
I3	3.09	1.26	−0.01	−1.01	0.81
I7	3.07	0.98	0.12	−0.16	0.75
I11	3.02	1.17	0.03	−0.71	0.74
I15	3.13	1.35	−0.14	−1.14	0.73
I19	2.95	1.28	−0.01	−1.03	0.68
I4	3.00	1.12	−0.05	−0.57	0.85
I8	3.10	1.30	−0.07	−1.02	0.76
I12	3.29	1.34	−0.18	−1.09	0.74
I16	2.85	1.36	0.14	−1.17	0.70
I20	3.10	1.26	−0.11	−0.89	0.69

### Exploratory Factor Analysis

The 20 items of PE-SPQ were submitted to exploratory factor analysis using the Unweighted Least Squares method. Sampling adequacy is supported by the KMO = 0.88, which measures sampling quality and the quality of the correlation matrices by the Bartlett significant test (χ2 = 4812.023, *p* < 0.001).

The results of the exploratory factor analysis by the Unweighted Least Squares method holding a Direct-Oblimin rotation with Kaiser normalization suggested the extraction of four factors that explain 64.8% of the variance. The first, the second, the third and the last factors explained 28.8% (Eigenvalue = 5.76), 18.10% (Eigenvalue = 3.61), 11.2% (Eigenvalue = 2.24) and 6.7% (Eigenvalue = 1.35), respectively.

**Figure 1:**
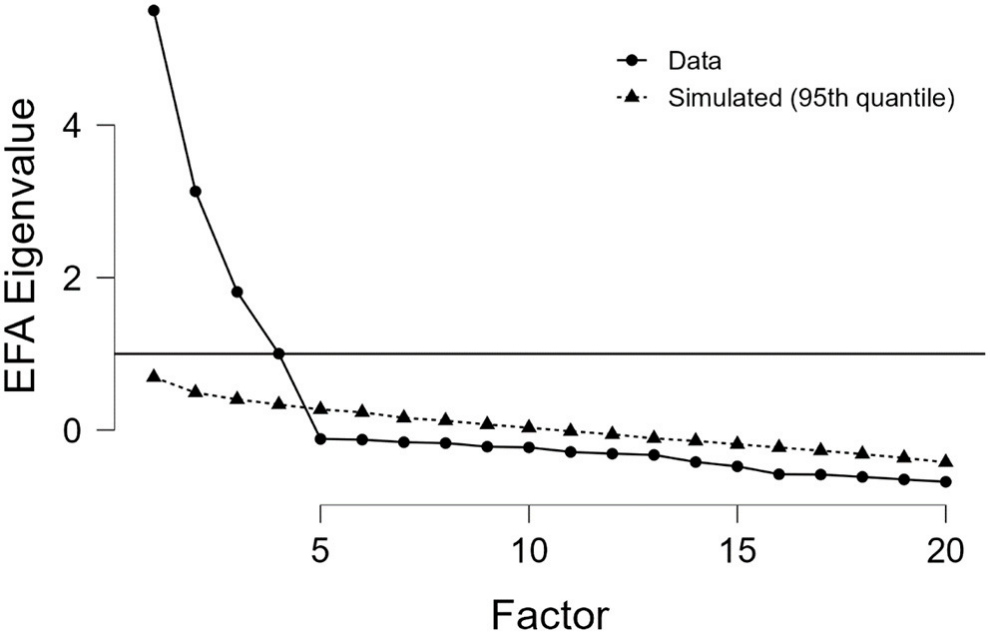
Scree plot of the PE-SPQ.

As shown in Figure 1, the purpose of the cut function is to select factors with Eigenvalue's superior to 1. The collected data and the simulated data (which are generated by the JASP software) showed a four-factor solution: the factors retained must be above the cut-off line perpendicular to the axis of the Eigenvalues (intersection for Eigenvalue = 1).

### Internal Consistency

The scale showed a good consistency coefficient for all components. For the DTT, McDonald's ω, Cronbach's α and Guttman's λ6 was 0.86, 0.86 and 0.83, respectively. A similar coefficient with a 0.86 value was demonstrated for DPT. In addition, for STT and SPT reliability coefficient are ranged between 0.89–0.91 and 0.92–0.94, respectively. These values indicated a good internal consistency for each of the four scales (see Table 2).

**Table 2:** Internal consistency of the PE-SPQ.

**Dimensions**	**McDonald's ω**	**Cronbach's α**	**Guttman's λ6**	**Average interitem correlation**	**Mean**	**Sd**
DTT	0.86	0.86	0.83	0.55	19.04	4.14
DPT	0.86	0.86	0.86	0.55	15.02	4.97
STT	0.91	0.91	0.89	0.66	15.27	5.18
SPT	0.94	0.93	0.92	0.74	15.33	5.70

*DPT, Deep Practical Task; DTT, Deep Theoretical Task; SPT, Surface Practical Task; STT, Surface Theoretical Task*.

### Confirmatory Factor Analysis

The univariate and descriptive statistics for the sample used in the confirmatory factor analysis are shown in Appendix Table 1. The multivariate Mardia's coefficient (4.56, z = 6.59, *p* < 0.01) indicated adequate multivariate normality.

**Figure 2:**
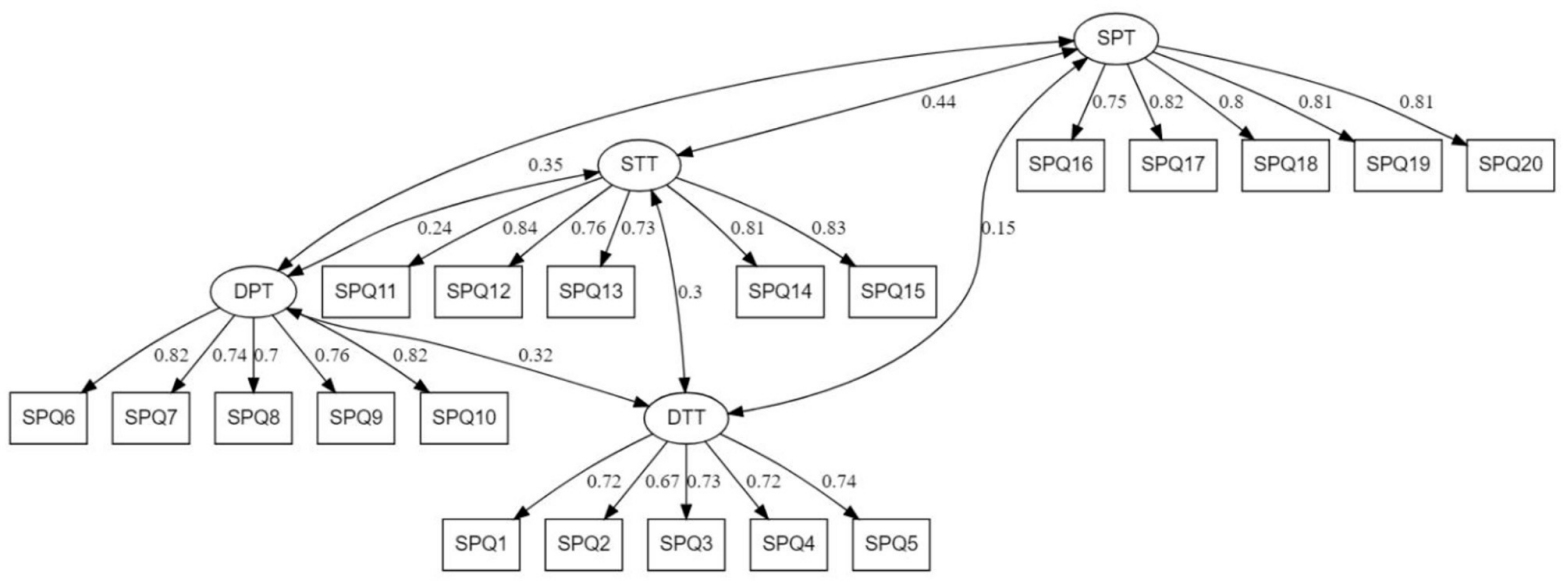
Confirmatory factor analysis of the PE-SPQ. χ2 = 466.47, df = 164, *p* < 0.001, χ2/df = 2.84, GFI = 0.92, AGFI = 0.90, TLI = 0.94, CFI = 0.95, SRMR = 0.046; RMSEA = 0.56 IC 90% [0.050–0.062]; DPT, Deep Practical Task; DTT, Deep Theoretical Task; SPT, Surface Practical Task; STT, Surface Theoretical Task.

The CFA results provided evidence for the four-factor structure of PE-SPQ. All the factorial weights of our items range from acceptable to excellent, as shown in Figure 2.

The results of the indices from the CFA showed a consistent first-order model with four factors, consistent with the theoretical model tested for the developed version of the scale (see Figure 2).

The χ 2 /df value is 2.84, the GFI index is 0.92, the AGFI index is 0.90. Moreover, RMSEA is 0.056, CFI is 0.95 and TLI is 0.94. Therefore, the theoretical model, which is a priori posed, was correctly reproduced by the empirically collected data.

### Convergent and Discriminant Validity

Convergent validity was examined by the average variance extracted (AVE). The AVE values were 0.51, 0.60, 0.62 and 0.64 for DTT, DPT, STT, SPT, respectively. These values demonstrated a good convergent validity or PE-SPQ. In addition, the AVE root mean square was lower than the correlations between factors (see Table 3). This confirms the discriminant validity of PE-SPQ.

**Table 3:** Pearson moment correlation between PE-SPQ and SPQ-2F.

	**DTT**	**DPT**	**STT**	**SPT**	**Deep**	**Surface**
DTT	0.72^£^					
DPT	0.27^**^	0.77^£^				
STT	0.26^**^	0.23^**^	0.79^£^			
SPT	0.12^**^	0.31^**^	0.40^**^	0.80^£^		
Deep	0.34^**^	0.12^**^	0.07	0.024	-	
Surface	−0.03	0.13^**^	0.21^**^	0.33^**^	0.39^**^	-

** *p < 0.01; £ Root mean square AVE*.

### Concurrent Scale Validity

To examine the concurrent validity of the scale, we performed the Pearson correlation of the four dimensions with the R-SPQ-2F scores. The results showed that DTT, and DPT are significantly and positively correlated with the Deep (r = 0.34 (*p* < 0.01), and r = 0.12 (*p* < 0.01), respectively. However, no links between STT and SPT with Deep was demonstrated (r = 0.07 and r = 0.024, respectively). No correlation was demonstrated between Surface and DTT. In addition, a weak correlation was found between Surface and DPT. Finally, significant correlations were affirmed on the one hand between Surface and STT (r = 0.21, *p* < 0.01), and on the other hand between Surface and SPT (r = 0.33, *p* < 0.01).

## Discussion

The aim of the present study is (a) to develop a new instrument: Physical Education Study Process Questionnaire (PE-SPQ) for measuring the study process in physical education and sports, and (b) to test the psychometric properties of the instrument in terms of factor structure, validity and reliability.

A four-factor scale was designed and empirically tested for physical education and sports students in Tunisia. The statistical results showed that the constructed measurement scale was appropriate for exploratory factor analysis. Empirical data for the 20 initially developed items were well-aligned with the proposed four factors of PE-SPQ. As a result, no item has been deleted. The results of the exploratory factor analysis suggest the presence of four factors that explain 64.8% of total variance. These outcomes are consolidated by the stability of the first-order solution which provided adequate fit indices. The results of the factorial structure in the two analyses are aligned with several studies which highlighting two distinct types of learning process. The distinct learning types suggest that an instrument for measuring learning process should be composed of items reflecting two factors [for example: Munchi et al. (85) and Hernández et al. (86)]. These results are similar to those of Biggs (87), whose study supported a two-factor structure through principal component analysis with varimax rotation. A pervious confirmatory factor analysis also supported the presence of two distinct components for the Surface and Deep subscales (87). However, other studies were unable confirm this structure [e.g., Stes et al. (28)].

Additionally, Justicia et al. (25) criticized the fact that in previous studies, each factor analysis was performed on the same sample. Although the factor loadings from the exploratory factor analysis supported the two-factor structure, mixed results were found in the fit indices from the confirmatory factor analysis [e.g., Fryer et al. (27)]. In contrast, Johnson et al. (32) found low fit indices using the weighted least squares (WLS) estimator. However, considerable improvement was confirmed using a maximum likelihood estimate.

In our study, we addressed the criticism of previous studies by conducting an exploratory factor analysis with one subset of the sample and a confirmatory factor analysis on another subset. The results of the confirmatory analysis supported the four-factor structure found in the exploratory analysis. Also, the factor analysis results from Immekus and Imbrie (26) did not support the factor structure of the original scale, suggesting instead an alternative four-factor model. The addition of the two factors DPT and SPT was similar to the original work, arguing that the hands-on learning process requires a deep component and a surface component. The present work is considered the first psychometric review of a scale that takes these factors into account.

Similarly, the reliability tests supported that the instrument is reliable among the four constructs. The confirmatory factor analysis showed the measurement robustness of the 20 items scale. These results were sustained by adequate convergent and discriminant validity and confirmed the robustness of the PE-SPQ as a new measure of study process in physical education. Additionally, the factors DTT and DPT are significantly and positively correlated with the Deep learning approach. Likewise, a weak correlation was found between Surface approach learning and DPT. These results demonstrated the concurrent validity of the PE-SPQ.

According to our best knowledge, no study has explored the validity and reliability of a questionnaire that measures the university study process in the context of physical education and sport. Similarly, the Biggs scale (24) widely used in the academic context has never been explored in this area.

In line with our findings regarding the four-factor structure of the questionnaire, Immekus and Imbrie (26) attempted a cross-validation of the two-factor questionnaire (R-SPQ-2F) on the basis of separate data from two samples of students attending a university in the United States (*n* = 1,490 and *n* = 1,533). The results of the factor analysis did not support the original factor structure of the scale, suggesting instead an alternative four-factor model of the data. Indeed, the integration of items that includes the physical dimension suggests a four-factor model. Similarly, the results of the multi-sample confirmatory factor analysis indicated that the parameters of the scaling model (e.g., factor loadings) were invariant in the independent samples.

The R-SPQ-2F scale was recently used in this study to evaluate learning approaches in 13 different subjects of four degrees (8, 88, 89). Item reliability analysis showed high consistency for the primary scales, but not for the secondary scales of the R-SPQ-2F questionnaire. In line with our results, a strong correlation between the deep and surface scale was observed. In another work, Khine and Afari (31) explored the reliability and validity of an adapted and translated Arabic version of the R-SPQ-2F questionnaire administered to students at a higher education institution in the United Arab Emirates. The analysis showed that the four factor of the Arabic version of the questionnaire was valid, as shown in both exploratory and confirmatory factor analysis. Similarly, the reliability of the tool was demonstrated by the classical Cronbach alpha index and the AVE (Average Variance Extracted) and CR (Composite Reliability).

Indeed, in different studies related to the cultural context, Biggs' questionnaire has been used to evaluate students' learning methods and its effectiveness has been evaluated in different socio-cultural contexts. In fact, the R-SPQ-2F questionnaire has been adapted to several languages (27–33) and has been tested in a variety of contextual areas such as medical students (90, 91), and chemistry students (92). For example, in medical studies, a deep learning approach has been demonstrated among students in Ghana (33), in Saudi Arabia (90) and in Malaysia (93). Similar results were obtained in business, computer science and engineering programs (94, 95). These results are different with those obtained among our participants. A variety of approaches are used by students in physical education. In fact, studies within the framework of physical education have not taken into account the specific characteristics of this subject (96). As a result, the physical component that is part of the curriculum and in assessments was overlooked (41, 42). By examining the curricula and the training of the students, except to find that the physical practice contributes significantly to the success among students in the institutes of physical education and sport. This systematically leads students to adopt different strategies. A significant mass of students can focus on physical practice to be successful if they have adequate athletic skills, while others focus on theoretical subjects to be successful.

To better explain the learning approach among our participants, we must explore the criteria for admission and success in these universities. For example, in the physical education and sports institutes in Tunisia, the official program of the LMD regime requires the student to capitalize a set of credits or an equivalence in notes of 50% regardless of the nature of the subjects. In other words, a student who has 10/20 as a general average successfully completed his university year. As a result, the student who excels in sports practice can succeed even with low marks in theoretical subjects. If this student has a good sports background as an example elite athlete, he may be successful in this discipline. While a middle-level student concentrates on theoretical subjects to fill in athletic deficiencies. These justifications can well explain the weak association between Deep approach with STT and SPT.

In Saudi Arabia, DA learning was generally associated with more hours of study and higher grades. In the same context and according to a study in the Netherlands (97), law students scored higher on DA than on SA, although many scored low on both learning approaches. Similarly, students of physical education have a very heavy learning content through practical and theoretical sessions. Because of this, these students may have different orientations and a different Grit (52).

Another study conducted in Australia with chemistry students using Biggs' original questionnaire found that DA learning was related to the assessment of learning objectives and the approach to learning and benefits was primarily affected by age (16). However, our study, was conducted among students without a great difference in age and academic years practice (3 years). Meanwhile, in Hong Kong, research using Biggs' unedited questionnaire and interviews revealed that DA learning was strongly correlated with age and negatively correlated with academic year (98).

However, according to studies conducted in Japan by Fryer et al. (27), validation of the Biggs questionnaire was performed against the DA and SA, but not against the subscales of the questionnaire, which showed distinct relationships that were not found in previous studies and could be explained by cultural motives. Work also conducted in China by Leung, Ginns et al. (99) found that learners adopted more intermediate approaches to learning, where memorization was a tool for understanding. Biggs' questionnaire was even used to test learners' progress in a subject area and the influence of the flipped lesson approach (100). Positive correlations were found between the DA and learners' learning outcomes (16, 100, 101). More studies are needs to examine PE-SPQ, in other cultural context.

As well as comparative studies of different question choice models that determine DA and SA learning in the Biggs questionnaire (102). The latter compared the findings of Biggs' work (24) in Hong Kong with those that evaluated the questionnaire in different contexts, [e.g., Spain (103); Japan (27); the United States (26, 102), Netherlands (28) or Norway (104)]. These studies have also indicated that there may be cultural differences that explain different results and have shown controversy regarding the grouping of test questions (26, 102, 104). In addition, another study comparing the results of students in Hong Kong and Sydney confirmed differences that could be attributed to cultural causes (99).

These studies analyzed the approach to learning in many contexts, but there is no detailed study that includes Tunisia's sociocultural conditions in a sport science learning environment. In the study, the approach of PE students was analyzed according to different degrees and levels, and the factors that influence their response were assessed.

We are aware that our research has some limitations. First, the instrument was only tested on a single population living in a single country. Second, multiple studies are needed to examine the study processes surveyed by the PE-SPQ across socio-demographic variables such as age and gender.

## Conclusion and Implications

The present study led to the design of the PE-SPQ questionnaire which is a new instrument adapted to the discipline. The tool was empirically tested in terms of its psychometric properties. The data confirmed the validity of the questionnaire in physical education and sport degrees. The measurement scale was stable, as evidenced by the exploratory factor analysis. Similarly, reliability tests proved that the scales derived from the instrument have good internal consistency. The confirmatory factor analysis showed the measurement robustness of the 20 items of the scale. Also, the four dimensions of the scale (DTT, DPT, STT, SPT) correlated with the deep and surface learning approach scales according to the expected pattern, which demonstrates its concurrent validity.

The findings of the study could be strengthened in future research by including more subjects from different disciplines and related subjects, and from other Tunisian universities. Future studies could confirm the number of factors and the associations of questions with the different scales of the R-SPQ-2F questionnaire and investigate the influence of pedagogical dynamics in the students' learning approaches in each subject.

## Data Availability Statement

The original contributions presented in the study are included in the article/Supplementary Material, further inquiries can be directed to the corresponding author.

## Ethics Statement

The studies involving human participants were reviewed and approved by the Ethics Committee of the Higher Institute of Sport and Physical Education of Kef, University of Jendouba, Jendouba, Tunisia, the Higher Institute of Sport and Physical Education of Sfax and the Higher Institute of Sport and Physical Education of Gafsa. The research was also approved by the Ethics Committee of the University of Jendouba and was undertaken in accordance with the legal standards of the Declaration of Helsinki 1964 and its corresponding amendments. The patients/participants provided their written informed consent to participate in this study.

## Author Contributions

AT conceived and designed the experiment. AT, NG, and NB collected and analyzed data. AT, NG, TB, NC, FA, and NB wrote the manuscript. All authors approved the submission.

## Conflict of Interest

The authors declare that the research was conducted in the absence of any commercial or financial relationships that could be construed as a potential conflict of interest.

## Publisher's Note

All claims expressed in this article are solely those of the authors and do not necessarily represent those of their affiliated organizations, or those of the publisher, the editors and the reviewers. Any product that may be evaluated in this article, or claim that may be made by its manufacturer, is not guaranteed or endorsed by the publisher.

## Abbreviations

AGFI: adjusted goodness-of-fit index
AVE: average variance extracted
CFI: comparative fit index
DA: deep approach
DPT: deep practical task
DTT: deep theoretical task
DWLS: Diagonally Weighted Least Squares
GFI: goodness-of-fit index
KMO: Kaiser-Meyer-Olkin
PE: physical education
PE-SPQ: physical education study process questionnaire
RMSEA: root mean square error of approximation
R-SPQ-2F: revised study process questionnaire-two-factors
SPT: surface practical task
SRMR: Standardized Root Mean Squared Residual
STT: surface theoretical task
TLI: Tucker-Lewis index.
