# Supplementary material for: Development and Preliminary Validation of the Physical Education-Study Process Questionnaire : Insights for Physical Education University Students
Source: Front Public Health. 2022 Mar 17;10:856167. doi: 10.3389/fpubh.2022.856167 (PMC8968756; doi:10.3389/fpubh.2022.856167)
Supplement: Supplementary file 1 [file Table_1.pdf]

السؤال	لا ينطبق أبدا	ينطبق بعض الأحيان	ينطبق نصف الأحيان	ينطبق كثير من الأحيان	ينطبق دائما
1.أجد أنّ الحصص التطبيقية للتربية البدنية في بعض الأحيان تعطيني شعور عميقا من الارتياح الشّخصي.					
2.لا بد لي من بذل جهد كبيرا بالحصص النظرية للتربية البدنية حتى أستطيع أن أشكّل استنتاجاتي الخاصة قبل أن أشعر بالارتياح.					
3. من أهدافي اجتياز الاختبارات التّطبيقيّة بأقلّ جهد مبذول.					
4. أدرس بجديه واجتهاد ما يعطى فقط خلال المحاضرات النظرية.					
5. أشعر أنّ أيّ موضوع متعلّق بالتربية البدنية، يمكن أن يصبح شيّقا جدّا عندما أتعمّق فيه بتوسّع.					
6. أجد أنّ معظم الدروس النظرية الجديدة مثيره للاهتمام، وكثيرا ما أنفق وقتا إضافيّا للحصول على مزيد من المعلومات عنها.					
7. لا أجد الحصص التطبيقية مهمه جدا، لذلك ألتزم بالحد الأدنى من العمل.					
8. أتمكن من دروسي النظرية بالتكرار، ومراجعتها عدة مرات الى أن احفظها عن ظهر قلب دون فهم محتواها.					
9. أجد أنّ الحصص التطبيقية الخاصة بالتربية البدنية في بعض الأحيان يمكن أن تكون مثيره وشيقة كمشاهدة مباراة ممتعة.					
10. أختبر نفسي في المواضيع الهامة والجديدة للتربية البدنية حتّى أفهمها تماما.					
11. يمكنني التعامل مع معظم عمليات التقييم بواسطة حفظ النقاط الرئيسية عن ظهر قلب بدل محاولة فهمها.					
12. بشكل عام أحدد دراستي فيما هو مطلوبُ تحديدا بالمواد النظرية دون فعل أي شيء اضافي.					
13. أبذل قصار جهدي في الحصص التطبيقية لأنّني أجد الموادّ مثيره للاهتمام ومشوّقه.					
14. أمضي الكثير من أوقات فراغي لمعرفة المزيد عن المواضيع الّتي نوقشت أثناء الحصص النظرية.					
15. أجد أنه لا فائدة من دراسة المواد التطبيقية بعمّق. فهذا يعدّ مشتّتا ومضيعة للوقت في حين ان كل ما أحتاج اليه هو التعرف على المواضيع بشكل عابر.					
16. أعتقد أنّه ليس من واجب طلبة التربية البدنية قضاء أوقات طويلة في استذكار مواضيع لا تدخل ضمن الامتحانات.					
17. عادة آتي إلى معظم حصص الدّروس التطبيقية وفي ذهني أسئلة حول عديد المواضيع أريد إجابات عليها.					
18. غالبا ما أهتمّ بمراجعة المعلومات المقدمة أثناء الحصص النظرية للتربية البدنية.					
19. لا أرى أيّ فائدة في التمكّن من أيّ درس من غير المحتمل أن يكون ضمن التقييم.					
20. أرى أنّ أفضل طريقه لاجتياز الامتحانات هو محاولة حفظ إجابات الأسئلة الّتي من المحتمل أن تأتي في الامتحان					

Deep Practical Task (DPT)<br>I1: I find that practical courses sometimes give me a deep sense of personal satisfaction.<br>I5: I feel that any topic related to physical education, can become very interesting when I delve into it extensively.<br>I9: I find that sometimes practical courses can be as exciting and interesting as watching an interesting game.<br>I13: I do my best in the practical courses because I find these sessions interesting.<br>I17: I usually come to most of the practical sessions with questions in my head on many topics that I want answers to.
Deep Theoretical Task (DTT)<br>I2: I have to do a lot of work in physical education class to be able to draw my own conclusions before I feel comfortable.<br>I6: I find most of the new theoretical courses very interesting, and I often spend extra time to get more information about them.<br>I10: I test myself on important and new topics in physical education until I fully understand them.<br>I14: I spend a lot of my free time learning more about the topics discussed during the theoretical courses.<br>I18: I am often interested in reviewing the information provided in the theoretical courses.
Surface Practical Task (SPT)<br>I3: One of my goals is to pass the practical exams with as little effort as possible.<br>I7: I don't find the practical courses very important, so I keep the work to a minimum.<br>I11: I can handle most assessments by rote learning the main points rather than trying to understand them.<br>I15: I find it unnecessary to study deep into the practical topics of physical education. It is a distraction and a waste of time when all I need is to familiarize myself with these topics in passing.<br>I19: I don't see the value in learning a lesson that will probably not be included in the assessment.
Surface Theoretical Task (STT)<br>I4: I study with seriousness and assiduity what is given only during the theoretical courses.<br>I8: I manage my theoretical courses by repetition, and go over them several times until I memorize them without understanding the content.<br>I12: In general, I limit my studies to what is required, that is, to the theoretical subjects without doing anything more.<br>I16: I don't think it is the duty of physical education students to spend long periods of time studying topics that are not included in the exams.<br>I20: I think the best way to do well on exams is to try to memorize the answers to questions that are likely to be asked on the exam.
